# Fatty Acid Accumulation and Resulting PPAR**α** Activation in Fibroblasts due to Trifunctional Protein Deficiency

**DOI:** 10.1155/2012/371691

**Published:** 2012-05-08

**Authors:** Masato Wakabayashi, Yuji Kamijo, Takero Nakajima, Naoki Tanaka, Eiko Sugiyama, Tian Yangyang, Takefumi Kimura, Toshifumi Aoyama

**Affiliations:** ^1^Department of Metabolic Regulation, Institute on Aging and Adaptation, Shinshu University Graduate School of Medicine, 3-1-1 Asahi, Matsumoto 390-8621, Japan; ^2^Department of Pharmacy, Nagano Red Cross Hospital, Nagano 380-8582, Japan; ^3^Department of Nephrology, Shinshu University School of Medicine, 3-1-1 Asahi, Matsumoto 390-8621, Japan; ^4^Department of Nutritional Science, Nagano Prefectural College, Nagano 380-8525, Japan; ^5^Department of Gastroenterology, Shinshu University School of Medicine, 3-1-1 Asahi, Matsumoto 390-8621, Japan

## Abstract

To examine fatty acid accumulation and its toxic effects in cells, we analyzed skin fibroblasts from six patients with mitochondrial trifunctional protein deficiency, who had abnormalities in the second through fourth reactions in fatty acid **β**-oxidation system. We found free fatty acid accumulation, enhanced three acyl-CoA dehydrogenases, catalyzing the first reaction in the **β**-oxidation system and being assumed to have normal activities in these patients, and PPAR**α** activation that was confirmed in the experiments using MK886, a PPAR**α** specific antagonist and fenofibrate, a PPAR**α** specific agonist. These novel findings suggest that the fatty acid accumulation and the resulting PPAR**α** activation are major causes of the increase in the **β**-oxidation ability as probable compensation for fatty acid metabolism in the patients' fibroblasts, and that enhanced cell proliferation and increased oxidative stress due to the PPAR**α** activation relate to the development of specific clinical features such as hypertrophic cardiomyopathy, slight hepatomegaly, and skeletal myopathy. Additionally, significant suppression of the PPAR**α** activation by means of MK886 treatment is assumed to provide a new method of treating this deficiency.

## 1. Introduction

The presence of an excessive level of fatty acids may cause significant toxicity in many organs and tissues. We recently examined the close relation between fatty acid toxicity and peroxisome proliferator-activated receptor (PPAR) functions. In some of our experiments, acute kidney injury was induced by albumin-overload nephropathy, in which PPAR*α* protected proximal tubular cells from acute toxicity induced by fatty acids bound to albumin [1]; furthermore, pretreatment with low-dose fibrates protected against the fatty-acid-induced renal tubule toxicity by counteracting PPAR*α* deterioration [2]. In our other experiments, hepatic steatosis and hepatocellular carcinoma in hepatitis C virus core protein transgenic mice were caused through fatty-acid-induced PPAR*α* activation [3, 4]. These *in vivo* experiments provided important results concerning fatty acid toxicity at the organ and tissue levels; however, the degree of the toxicity differed greatly, even among the same types of cells. We, therefore, undertook several experiments using cultured cells to elucidate the detailed mechanisms in the cell toxicity. We adopted fibroblasts from patients having a certain abnormality in the mitochondrial fatty acid *β*-oxidation system.

 Trifunctional protein (TP), catalyzing fatty acid *β*-oxidation in mitochondria, is a multienzyme complex composed of four molecules of the *α*-subunit containing the enoyl-CoA hydratase and 3-hydroxyacyl-CoA dehydrogenase domains and four molecules of the *β*-subunit containing the 3-ketoacyl-CoA thiolase domain. An inborn error of this enzyme complex can cause sudden infant death syndrome, hepatomegaly accompanying fat accumulation, hepatic encephalopathy, skeletal myopathy, or hypertrophic cardiomyopathy with rather high frequency. This deficiency is classified into two different phenotypes: one represents the existence of both subunits and the lack of only the 3-hydroxyacyl-CoA dehydrogenase activity and the other represents the absence of both subunits and the lack of the three activities, although the clinical features of these two phenotypes are similar [5–7].

## 2. Materials and Methods

### 2.1. Chemicals

MK886, a PPAR*α*-specific antagonist [8] and fenofibrate were obtained from Wako Pure Chemical (Osaka, Japan) and Sigma Chemical Company (St. Louis, MO, USA), respectively.

### 2.2. Source of the Cells and Culture Method

The case histories of the TP patients were reported elsewhere [5–7]. Skin fibroblasts were collected from the patients [5, 9, 10] and cultured in the medium containing Dulbecco's modified Eagle's medium, 10% (v/v) fetal calf serum, 0.1 mM nonessential amino acids, 1 X antibiotic-antimycotic solution (Invitrogen Life Technologies Corp., Carlsbad, CA, USA) and 4.5 mg D-glucose/mL.

### 2.3. Acyl-CoA Dehydrogenase Activity

About 1-2 mg of fibroblasts was suspended in 150 *μ*L of solution containing 67 mM potassium phosphate (pH 7.5), 200 mM sodium chloride, and 0.6% (w/v) Triton X-100. The suspension was gently sonicated, and the solution was centrifuged at 3,000 ×g for 5 min. Fifty *μ*L of the supernatant fraction was mixed with a solution containing 67 mM potassium phosphate (pH 7.5), 40–50 *μ*M palmitoyl-CoA (or octanoyl-CoA), and 0.4 *μ*M electron transfer flavoprotein, in a final volume of 1.5 mL. The mixture without the electron transfer flavoprotein was preincubated for 2 min at 37°C with gentle bubbling of nitrogen gas to exclude oxygen. The reaction was started by addition of electron transfer flavoprotein, and run under nitrogen gas. Electron transfer flavoprotein reduction was measured by using a fluorometer (Hitachi F-2000) with excitation at 342 nm and emission at 496 nm [11]. The activities in fibroblasts were also measured by the method coupling with ferricenium ion [12] in order to confirm them.

### 2.4. Immunoblot Analysis

Protein concentrations were measured colorimetrically with a BCA Protein Assay kit (Pierce Biotechnology Inc., Rockford, IL, USA). Whole-cell lysates (60 *μ*g protein) were subjected to 10% SDS-polyacrylamide gel electrophoresis [13]. After electrophoresis, the proteins were transferred to nitrocellulose membranes, which were incubated with the primary antibody and then with alkaline phosphatase-conjugated goat anti-rabbit IgG. Antibodies against VLCAD, LCAD, and MCAD were described previously [14, 15]. The band of actin was used as the loading control. Band intensities were measured densitometrically, normalized to those of actin, and then expressed as fold changes relative to the averaged value of the three control fibroblasts.

### 2.5. Analysis of mRNA

Total RNA was extracted using an RNeasy Mini Kit (QIAGEN, Hilden, Germany), and samples of 2 *μ*g of RNA were reverse transcribed using oligo-dT primers and SuperScript II reverse transcriptase (Invitrogen Life Technologies Corp.). Levels of mRNA were quantified by real-time polymerase chain reaction using a SYBR Premix Ex Taq II (Takara Bio, Otsu, Japan) on a Thermal Cycler Dice TP800 system (Takara Bio) [3, 16]. Specific primers were designed by Primer Express software (Applied Biosystems, Foster City, CA, USA): 5′-GAGCCACGGACTTCCAGATA-3′ and 5′-GCATTCATCTGTCACCTTCCA-3′ for the VLCAD gene; 5′-TCACTCAGAATGGGAGAAAGC-3′ and 5′-CTCCAATTCCACCAAGATGCT-3′ for the LCAD gene; 5′-TAACCAACGGAGGAAAAGCT-3′ and 5′-CTGCTTCCACAATGAATCCA-3′ for the MCAD gene; 5′-GTGAAATCGGGACCCATAAG-3′ and 5′-CGATGGTTGTCCATTTTGAG-3′ for the peroxisomal acyl-CoA oxidase gene; 5′-CCATTCGATCTCACCAAGGT-3′ and 5′-GGATTCCGGTTTAAGACCAGTT-3′ for the catalase gene; 5′-GGAGGGAGCTGACTGATACACT-3′ and 5′-TCAGCAGGTTGGCAATCTC-3′ for the c-Fos gene; 5′-GGACTATCCTGCTGCCAAGA-3′ and 5′-CTGGTGCATTTTCGGTTGTT-3′ for the c-Myc gene; 5′-CACTGGTGGTCCATGAAAAAG-3′ and 5′-ACTTCCAGCGTTTCCTGTCT-3′ for the Cu, Zn-superoxide dismutase gene; 5′-CCGAGAAGCTGTGCATCTACA-3′ and 5′-GGTTCCACTTGAGCTTGTTCA-3′ for the cyclin D1 gene; 5′-TGTATGGAAGAGCCCAGATTC-3′ and 5′-GCACAGTACAGGCACAAAGGT-3′ for the NADPH oxidase 4 gene; 5′-GGCGTGAACCTCACCAGTAT-3′ and 5′-GCGTTATCTTCGGCCCTTAG-3′ for the proliferating cell nuclear antigen gene; 5′-CCTCAAGATCATCAGCAATGC-3′ and 5′-GGTCATGAGTCCTTCCACGAT-3′ for the GAPDH gene. The mRNA levels of glyceraldehyde-3-phosphate dehydrogenase (GAPDH) were used as an internal control. Measurements of mRNA levels were normalized to those of GAPDH and then expressed as fold changes relative to the averaged value of the three control fibroblasts.

### 2.6. Assays for DNA Binding Activity of PPARs

The DNA-binding activity of PPAR*α*, PPAR*β*, and PPAR*γ* was determined using the PPAR*α*, PPAR*β*, and PPAR*γ* Transcription Factor Assay kits (Cayman Chemical, Ann Arbor, MI, USA) [17–19], respectively. These assays are based on an enzyme-linked immunosorbent assay using PPAR response element- (PPRE-) immobilized microplates and specific PPAR antibodies, thus offering similar results to those from the conventional radioactive electrophoretic mobility shift assay. DNA-binding assays were carried out according to the manufacturer's instructions using whole-cell lysates (100 *μ*g protein). Results are expressed as fold changes relative to the averaged value of the three control fibroblasts.

### 2.7. Analyses of TG and FFA

To determine the content of triglycerides (TGs) and free fatty acids (FFAs), lipids were extracted according to a method reported by Hara and Radin [20]. TG and FFA were measured with Triglyceride *E*-test kit and an NEFA *C*-test kit (Wako Pure Chemical, Osaka, Japan), respectively.

### 2.8. Statistical Analysis

All data are expressed as mean ± standard deviation (SD). Statistical analysis was performed using one-way ANOVA with Bonferroni correction (SPSS Statistics 17.0; SPSS Inc, Chicago, IL, USA). Correlation coefficients were calculated using Spearman's rank correlation analysis. A probability value of less than 0.05 was considered to be statistically significant.

## 3. Results

### 3.1. Acyl-CoA Dehydrogenase Activity and the Content of TG/FFA in Fibroblasts

**Figure 1:**
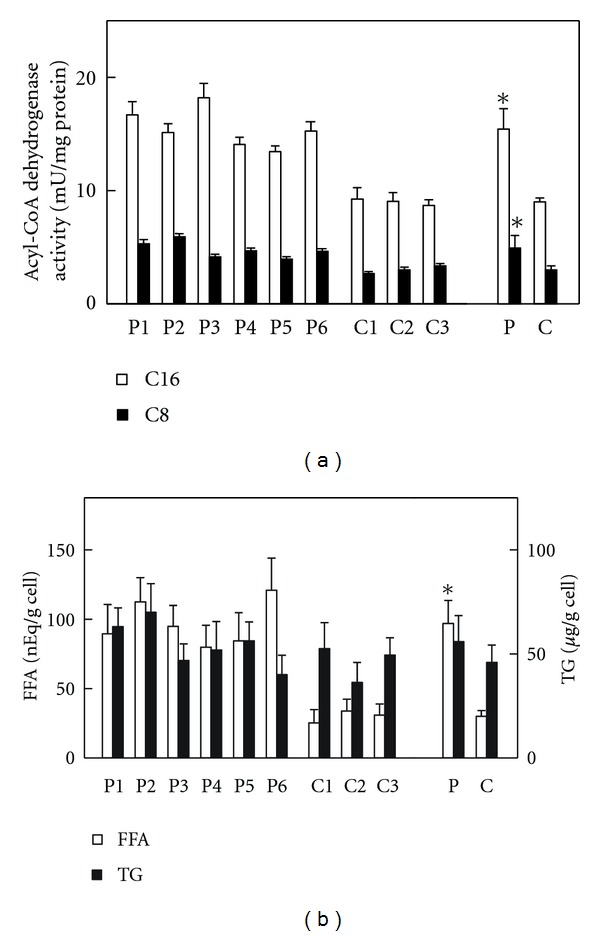
Acyl-CoA dehydrogenase activity and FFA/TG contents in fibroblasts. Assay methods were, respectively, described in Section 2. (a) Indicates palmitoyl-CoA (open bar, C16) and octanoyl-CoA (closed bar, C8) dehydrogenase activities, respectively. (b) Indicates FFA (open bar) and TG (closed bar) contents, respectively. P1–P6, individual patient's fibroblast; C1–C3, individual control fibroblast; P, means ± SD in six patients' fibroblasts; C, means ± SD in three control fibroblasts. *P < 0.05 versus controls.

Six strains of skin fibroblasts were prepared from the individual TP patients, as well as the three strains from the healthy adult men as described in Section 2. The reproductive rate of these fibroblasts was similar in all strains. TP deficiency is based on abnormalities in the second through fourth reactions in the mitochondrial fatty acid *β*-oxidation system; therefore, acyl-CoA dehydrogenase, catalyzing the first reaction in the *β*-oxidation system, was assumed to be normal in the patients' fibroblasts. Additionally, increased levels of FFA/TG due to the impaired *β*-oxidation ability in these fibroblasts were expected. Thus, these parameters were analyzed first. Palmitoyl-CoA and octanoyl-CoA dehydrogenase activities in the patients' fibroblasts were 1.72- and 1.64-fold higher than those in the control fibroblasts, respectively, (Figure 1(a)). FFA content in the patients' fibroblasts was 3.2-fold higher than that in the control fibroblasts, while TG levels were similar in both fibroblasts. These FFA and TG levels were much lower than those in human serum (Figure 1(b)).

### 3.2. Expression of Three Acyl-CoA Dehydrogenases

**Figure 2:**
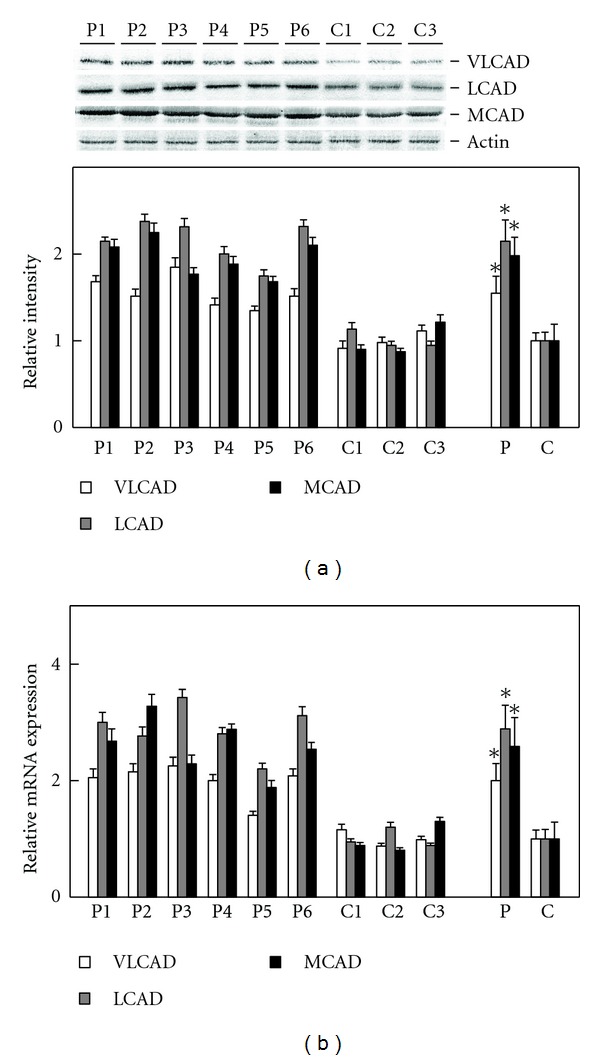
Expression levels of three Species of acyl-CoA dehydrogenase. Assay methods were, respectively, described in Section 2. (a) Shows relative quantification of expression levels of three acyl-CoA dehydrogenases. Upper panel indicates protein bands in immunoblot analysis. The band of actin was used as the loading control. Lower panel indicates relative protein amounts obtained by immunoblot and densitometric analyses. (b) Shows relative mRNA expression. Open bar, VLCAD; gray bar, LCAD; closed bar, MCAD. P1–P6, individual patient's fibroblast; C1–C3, individual control fibroblast; P, means ± SD in six patients' fibroblasts; C, means ± SD in three control fibroblasts. *P < 0.05, versus controls.

Palmitoyl-CoA and octanoyl-CoA dehydrogenation are catalyzed by three forms of acyl-CoA dehydrogenase; therefore, their expression levels were examined. The protein levels of VLCAD, LCAD, and MCAD in the patients' fibroblasts were 1.55-, 2.15-, and 1.97-fold higher than those in the control fibroblasts, respectively, (Figure 2(a)). The mRNA contents of VLCAD, LCAD, and MCAD in the patients' fibroblasts were 2.00-, 2.92-, and 2.63-fold higher than those in the control fibroblasts, respectively, (Figure 2(b)). These data were consistent with the observations shown in Figure 1(a). The simultaneous increases in the expression levels of the three forms of acyl-CoA dehydrogenase strongly suggested the presence of PPAR*α* activation in the patients' fibroblasts, since the three forms are known as PPAR*α* target gene products [15]. The presence of PPAR*α* activation was thereby examined in detail.

### 3.3. Assays for DNA-Binding Activity of PPARs

**Figure 3:**
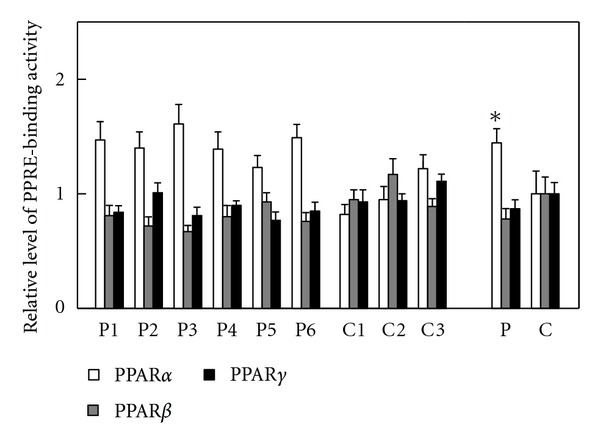
PPRE-binding activity. Assay methods were described in Section 2. Open bar, PPAR*α*; gray bar, PPAR*β*; closed bar, PPAR*γ*. P1–P6, individual patient's fibroblast; C1–C3, individual control fibroblast; P, means ± SD in six patients' fibroblasts; C, means ± SD in three control fibroblasts. *P < 0.05, versus controls.

Immunoblot analysis using whole-cell lysates from the fibroblasts and specific antibodies was performed and provided very faint bands for PPAR*β* and no bands for PPAR*α* and PPAR*γ*. mRNA analysis was also done as described in Section 2, and indicated that the PPAR*α*, *β*, and *γ* mRNAs were 10^−6^~10^−4^ levels for GAPDH mRNA in the fibroblasts, meaning that the data from the immunoblot and mRNA analyses were unreliable for detecting PPAR activation. The PPRE-binding assay was then done, which demonstrated an increase of PPRE-binding activity only for PPAR*α* in the whole-cell lysates from the patients' fibroblasts (Figure 3). These data supported the presence of PPAR*α* activation in the patients' fibroblasts.

### 3.4. Treatments with MK886 and Fenofibrate

**Figure 4:**
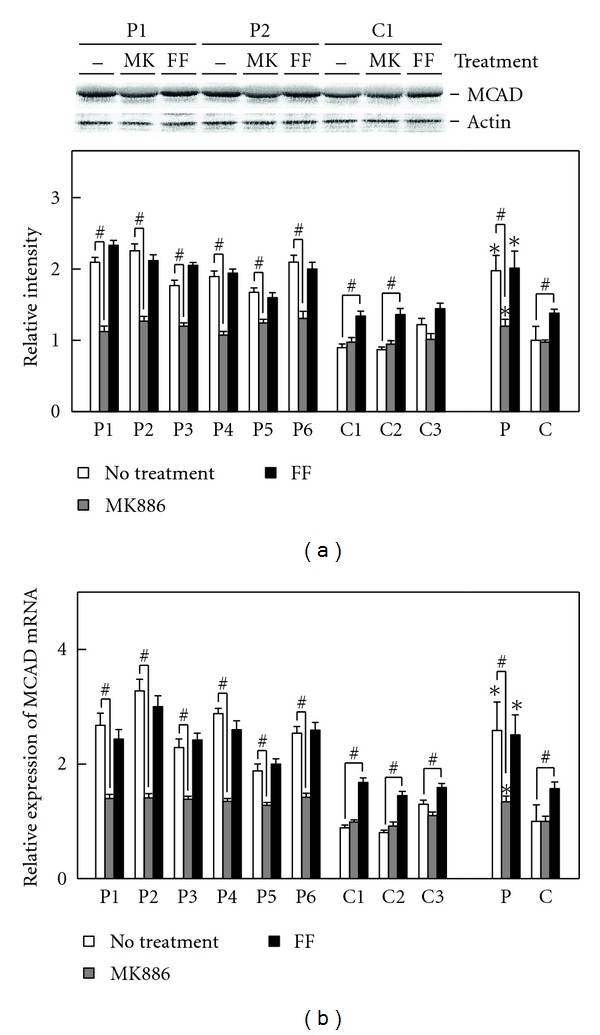
Effects of the MK886 or fenofibrate treatment on MCAD expression. The fibroblasts were plated in dishes and allowed to grow to 80% confluence. MK886 (30 *μ*M final concentration) and fenofibrate (200 *μ*M final concentration) were added to cell culture media, respectively. Both chemicals were dissolved in DMSO, and the final concentration of DMSO in media was maintained at 0.05% (v/v) in all cases. After 6 h, fibroblasts were harvested and used to analyze MCAD expression. (a) Shows relative quantification of expression level of MCAD protein. Upper panel indicates protein bands in immunoblot analysis. The band of actin was used as the loading control. Protein bands of two patients (P1 and P2) and a control (C1) are shown due to space limitation. Lower panel indicates relative protein amounts obtained by immunoblot and densitometric analyses. (b) Shows relative mRNA expression. Open bar, no treatment; gray bar, MK886 treatment; closed bar, fenofibrate (FF) treatment. P1–P6, individual patient's fibroblast; C1–C3, individual control fibroblast; P, means ± SD in six patients' fibroblasts; C, means ± SD in three control fibroblasts. *P < 0.05, versus controls; ^#^ *P* < 0.05, no treatment versus MK886 or fenofibrate treatment.

To confirm the appearance of PPAR*α* activation in the patients' fibroblasts, the fibroblasts were treated with MK886, a PPAR*α*-specific antagonist and fenofibrate, a PPAR*α* specific agonist, respectively. The expression level of MCAD, a representative PPAR*α* target gene product, was investigated. In the patients' fibroblasts, the MK886 treatment evidently reduced MCAD expression both in the protein and mRNA levels, and the fenofibrate treatment left this expression unchanged. In the control fibroblasts, the MK886 treatment did not affect this expression, and the fenofibrate treatment increased it both in the protein and mRNA levels (Figure 4). These data demonstrated that a considerable level of PPAR*α* activation constitutively functioned in the patients' fibroblasts.

## 4. Discussion

This study demonstrated the occurrence of FFA accumulation, increased palmitoyl-CoA and octanoyl-CoA dehydrogenase activities, coordinated enhancement in the expression of three acyl-CoA dehydrogenases, a significant increase of PPRE-binding activity only for PPAR*α*, and reduced MCAD expression as a result of PPAR*α*-specific antagonist treatment in all of the fibroblasts from six patients with TP deficiency who had abnormalities in the second through fourth reactions in the mitochondrial fatty acid *β*-oxidation system. These results demonstrated that a considerable level of PPAR*α* activation constitutively functioned in the patients' fibroblasts, in which FFA seemed to act as endogenous ligands toward PPAR*α* as reported elsewhere [21–23]. FFA seems to work not toxically but protectively in the patients' fibroblasts, since the FFA accumulation and the resulting PPAR*α* activation probably compensated for the impaired fatty acid metabolism in the patients' fibroblasts. It would be interesting to investigate whether this compensation appears in the patients' liver and heart, where considerable increases of TG/FFA and much higher levels of PPAR*α* expression are expected [3, 24, 25]. From this viewpoint, the results obtained by using the patients' fibroblasts in the current study are useful for understanding the PPAR*α* function.

This PPAR*α* activation might induce cell proliferation in the patients' fibroblasts. To examine it, the mRNA levels with several oncogene products and cell cycle regulators were analyzed. The mRNA levels in the patients' fibroblasts were 2.5 ± 0.5-fold for c-Fos, 3.0 ± 0.6-fold for c-Myc, 2.4 ± 0.7-fold for cyclin D1, and 2.1 ± 0.3-fold for proliferating cell nuclear antigen, which are all known as possible PPAR*α* target gene products [3], when compared with those in the control fibroblasts. These results suggest the presence of promoted cell proliferation in the patients' fibroblasts, which appears to be helpful for elucidating the mechanisms of hypertrophic cardiomyopathy and hepatomegaly that occur in TP-deficient patients. Additionally, this work described increased oxidative stress in the patients' fibroblasts. Biochemical analysis measuring 4-hydroxyalkenals and malondialdehyde with the use of an LPO-586 kit demonstrated 2.2 ± 0.2 times greater lipid peroxides contents in the patients' fibroblasts than in the control fibroblasts, implying enhanced levels of oxidative stresses in the former fibroblasts. This finding was consistent with the results of mRNA analysis: the mRNA levels in the patients' fibroblasts were 2.7 ± 0.3-fold for peroxisomal acyl-CoA oxidase, which is known as a representative PPAR*α* target gene product [3, 15], 1.3 ± 0.4-fold for catalase, 0.9 ± 0.3-fold for Cu, Zn-superoxide dismutase, and 1.1 ± 0.4-fold for NADPH oxidase 4, when compared with those in the control fibroblasts. This increased oxidative stress might help to elucidate the mechanisms of skeletal muscle weakness and hepatic encephalopathy, which occur in many TP-deficient patients. The FFA accumulation and the resulting PPAR*α* activation seem to exert not protective but toxic effects on the patients' fibroblasts, since the activation aggravates intracellular circumstances by increasing oxidative stresses and promoting cell proliferation, which counteracts the protective role mentioned above of compensating for the impaired fatty acid metabolism in the patients' fibroblasts.

 Additionally, this research pointed out the significant suppression of the PPAR*α* activation by the MK886 treatment, which might be useful to eliminate the toxic effects of the activation. Thus, the MK886 treatment together with the administration of glucose or sucrose to supply energy might offer a new method for treating this deficiency.

By the way, short-chain (SCAD), medium-chain, long-chain, and very-long-chain acyl-CoA dehydrogenases are known to catalyze the first reaction in the mitochondrial *β*-oxidation system. Among the four isozymes, the presence of SCAD, MCAD, and VLCAD deficiencies has been reported. Patients with SCAD deficiency occasionally represented skeletal muscle weakness and developmental delay [26, 27], and those with MCAD deficiency frequently exhibited fasting intolerance and hypoketotic hypoglycemia [28, 29], which is rather dissimilar to the clinical features of patients with TP deficiency. On the other hand, patients with VLCAD deficiency frequently presented with fasting coma, skeletal muscle weakness, skeletal myopathy, hypertrophic cardiomyopathy, cardiomegaly, and slight hepatomegaly with fat accumulation [30–32], conditions which are similar to the clinical features of patients with TP deficiency. This similarity may depend on the PPAR*α* activation induced by accumulated long-chain fatty acids and their derivatives. A future study using fibroblasts from patients with VLCAD deficiency is expected to confirm the mechanisms mentioned in this paper.

## Abbreviations

FFA: Free fatty acids
GAPDH: Glyceraldehyde-3-phosphate dehydrogenase
LCAD: Long-chain acyl-CoA dehydrogenase
MCAD: Medium-chain acyl-CoA dehydrogenase
PPAR: Peroxisome proliferator-activated receptor
PPRE: PPAR response element
TG: Triglycerides
TP: Trifunctional protein
VLCAD: Very-long-chain acyl-CoA dehydrogenase.
